# Метаболический фенотип взрослых потомков мышей, полученных при разных вариантах эмбриональных пересадок

**DOI:** 10.18699/VJ20.671

**Published:** 2020-11

**Authors:** M.V. Anisimova, Ya. Gong, N.S. Yudin, Yu.M. Moshkin, L.A. Gerlinskaya

**Affiliations:** Institute of Cytology and Genetics of Siberian Branch of the Russian Academy of Sciences, Novosibirsk, Russia; Novosibirsk State University, Novosibirsk, Russia; Institute of Cytology and Genetics of Siberian Branch of the Russian Academy of Sciences, Novosibirsk, Russia; Institute of Cytology and Genetics of Siberian Branch of the Russian Academy of Sciences, Novosibirsk, Russia; Institute of Cytology and Genetics of Siberian Branch of the Russian Academy of Sciences, Novosibirsk, Russia

**Keywords:** embryo transfer, mature offspring, metabolic phenotype, body composition, glucose tolerance test, пересадки эмбрионов, половозрелые потомки, метаболический фенотип, состав тела, глюкозотолерантный тест

## Abstract

Вспомогательные репродуктивные технологии (ВРТ) занимают все более заметное место в репро- дуктологии. Кроме того, в развитых странах ВРТ обеспечивают воспроизводство более 50 % крупного рогато- го скота, а в коллекциях генетических линий лабораторных животных являются неотъемлемым компонентом криоархивирования и редеривации. Процедуры ВРТ включают развитие ранних эмбрионов вне материнского организма и высокую вероятность неполной синхронизации физиологического состояния суррогатной матери и пересаживаемых эмбрионов. Поскольку все это происходит на стадии наибольшей восприимчивости заро- дышей к эпигенетическому перепрограммированию, то полный цикл ВРТ и его отдельные составляющие могут приводить к устойчивым фенотипическим изменениям потомков. Данное влияние подтверждают исследования морфофункциональных характеристик половозрелых потомков мышей аутбредной линии CD1, полученных с использованием разных вариантов трансплантаций ранних эмбрионов. Сравнительные исследования массы и состава тела, базального уровня глюкозы и реакции на глюкозную нагрузку (глюкозотолерантный тест) выполне- ны на половозрелых самцах и самках, потомках матерей, не подвергавшихся экспериментальным воздействиям в период беременности (группа контроля); двухклеточных эмбрионах, вымытых у беременных самок, после ин- кубирования до стадии бластоцист и пересаженных суррогатным матерям (группа 2 кл. – бл.); при пересадках двухклеточных эмбрионов (группа 2 кл. – 2 кл.) и бластоцист (группа Бл. – бл.) сразу после вымывания. Во всех экспериментах эмбрионы пересаживали вынашивающим самкам той же линии. Установлено, что половозрелые потомки, полученные при всех вариантах пересадок, характеризуются большим по сравнению с контрольными особями относительным содержанием жира и, соответственно, меньшей тощей массой тела. Этот эффект был вы- ражен сильнее у самок, чем у самцов. В отличие от состава тела пересадки эмбрионов в большей степени влияли на базальную концентрацию глюкозы и показатели глюкозотолерантного теста у самцов, чем у самок. При этом потомки групп 2 кл. – 2 кл. и 2 кл. – бл. характеризовались более высокой толерантностью к нагрузке глюкозой по сравнению с контрольной группой и группой Бл. – бл. Устойчивые отклонения состава тела и показателей гомеостаза глюкозы, выявленные у потомков при разных вариантах эмбриотрансплантаций, свидетельствуют о фенотипической значимости процедур, используемых при вспомогательных репродуктивных технологиях.

## Abstract (en)

Assisted reproductive technologies (ART) increasingly occupy the study of human reproduction. In addition, in developed countries they contribute to breeding of more than 50 % of cattle. In the management of collections of genetic lines of laboratory animals, these technologies are obligatory components of cryopreservation and rederivation. ART procedures include the development of early embryos outside the mother’s body and the high probability of incomplete synchronization of the physiological state of the surrogate mother and transplanted embryos. Since all this occurs at the stage of the highest susceptibility of embryos to epigenetic reprogramming, the full cycle of ART and its individual components can lead to stable phenotypic changes in the offspring. Their reality is confirmed by studies of the morphological and functional characteristics of sexually mature offspring of CD1 outbred mice, obtained using different variants of early embryo transplantation. Comparative studies of body mass and body composition, basal glucose level and response to glucose load (glucose-tolerance test – GTT) have been done on sexually mature males and females. Animals were separated in 4 groups according to the variant of embryo transplantation: group (control) – natural mating; group (2cl-bl) – incubation of 2-cell up to blastocysts; group (2cl-2cl) – removal and transplantation of the 2-cell embryo without incubation; group (Bl-bl) removal and transplantation of the blastocysts without incubation. All embryos were transplanted to recipient females of the same line. It was found that sexually mature offspring obtained with all variants of transplantations had a higher relative fat content and, correspondingly, lower lean mass compared to the control. This effect was more pronounced in females than in males. Unlike body compositions, embryo transplantations had a greater effect on basal glucose concentration and GTT in males than in females. In this case, the offspring of the 2cl-2cl and 2cl-bl groups were characterized by a higher tolerance to glucose load (GTT) compared with the control and the Bl-bl group. Stable deviations of body compositions and glucose homeostasis indices detected in experimental groups of progenies indicate the phenotypic significance of the embryo transplantations per se.

## Введение

В мире более 45 млн брачных пар сталкиваются с бес- плодием. Преодолеть эту проблему помогают вспомога- тельные репродуктивные технологии: экстракорпоральное оплодотворение (ЭКО) и внутриклеточная инъекция сперматозоида (intracytoplasmic sperm injection – ИКСИ). Со времени первого успешного применения ВРТ в кли- нической практике (1978 г.) число детей, рожденных с помощью ЭКО, возросло до 7 млн во всем мире. Сегодня в развитых странах ~1 % детей рождаются методом ЭКО (International Committee…, 2012). Большинство этих детей относят к категории здоровых (Davies et al., 2012), хотя в некоторых исследованиях отмечаются потенци- альные риски патологий беременности и новорожденных (Templeton, 2000; Hansen et al., 2013). При проведении ЭКО повышается риск осложнений беременности (Romundstad et al., 2008; Esh-Broder et al., 2011; Chen et al., 2015), среди которых аномальный рост плаценты, пери- натальная смертность, преждевременные роды и низкий вес при рождении (Helmerhorst et al., 2004; Ceelen et al., 2008; Rinaudo, Lamb, 2008; Haavaldsen et al., 2012). Дети, зачатые с помощью ЭКО, в подростковом возрасте демон- стрируют статистически значимые различия в динамике роста (Ceelen et al., 2009), отложении жира (Ceelen et al., 2007), уровнях артериального давления и концентрации глюкозы в крови (Ceelen et al., 2008).

При использовании ЭКО у лабораторных животных и крупного рогатого скота наблюдается внутриутробное ограничение роста на ранних сроках беременности, за которым следуют ускоренные темпы развития плода от средней до поздней беременности, что коррелирует с увеличением роста плаценты. Исследования на коровах и овцах дополнительно показывают, что потомки, получен- ные методом ЭКО, демонстрируют уникальный фенотип, так называемый синдром крупного потомства (Young et al., 1998; Sinclair et al., 2000; Farin et al., 2006).

Успешность преимплантационного развития обеспечи- вается строго скоординированными физиологическими и эпигенетическими трансформациями в период развития от зиготы до бластоцисты. Поддержка здоровой беремен- ности обеспечивается многочисленными материнскими факторами, контролирующими процессы созревания гамет, оплодотворение, доимплантационное развитие и им- плантацию бластоцист. Согласно гипотезе Бакера, небла- гоприятные условия материнской среды играют ведущую роль в развитии отклонений в период внутриутробного развития и, как следствие, формировании физиологи- ческого и метаболического фенотипа новорожденных, ассоциированного с увеличением риска хронических за- болеваний во взрослом возрасте (Barker, 2007). Метабо- лические потребности развивающегося эмбриона зависят от стадии клеточного деления и удовлетворяются за счет гуморального состава внутриматочной среды, включая питательные вещества и факторы роста, которая меняется по мере того, как эмбрион перемещается из яйцевода в матку (Leese, 2012). Важно отметить, что динамичность гуморального окружения практически отсутствует при культивировании эмбрионов in vitro.

Риски долговременных неблагоприятных последствий процедур ЭКО зависят от многих переменных, таких как качество и способ получения ооцитов; метод фер- тилизации in vitro (ЭКО / ИКСИ); состав культуральной среды; физические факторы окружающей среды (СО2 /О2, температура, влажность); продолжительность развития в условиях in vitro. Кроме того, на беременность и онтоге- нез влияет синхронизация степени развития эмбрионов и морфофункционального состояния организма вынаши- вающей матери. Плодово-материнская синхронизация во многом зависит от стадии эмбриогенеза и трансплантации зародышей в яйцевод или матку.

В данной работе показаны долговременные последствия инкубирования in vitro двухклеточных эмбрионов до ста- дии бластоцист и пересадок либо двухклеточных эмбрио- нов в яйцевод, либо бластоцист в матку на метаболический фенотип взрослых потомков. Эти варианты пересадок моделируют ВРТ при: а) редеривации и криоархивиро- вании двухклеточных эмбрионов с последующей пере- садкой в яйцевод; б) размножении сельскохозяйственных животных на основе криоархивированных бластоцист; в) выполнении ЭКО или ИКСИ в клинической практике, которые включают оплодотворение in vitro, инкубирова- ние до стадии бластоцисты и пересадку суррогатной матери. Результаты показали, что все варианты пересадок влияют на метаболический фенотип половозрелых потомков, что проявляется в статистически значимых из- менениях состава тела и толерантности к глюкозе. Кроме того, это исследование подтвердило важность использо- вания зачатых in vivo эмбрионов с последующей пере- садкой суррогатным реципиентам в качестве надлежащих контролей для дальнейшего изучения долговременных фенотипических эффектов экстракорпорального оплодо- творения.

## Материалы и методы

**Условия содержания и животные**

Исследование выполнено в ЦКП «Центр генетических ресурсов лабораторных животных» ИЦиГ СО РАН (RFME FI61914X0005 и RFMEFI62114X0010). Мышей аутбред- ной линии CD1 содержали в контролируемых условиях среды: фотопериоде 14С:10Т, при температуре 22–24 °С и влажности 40–50 %. В качестве подстилочного мате- риала использовали обеспыленные березовые гранулы (ООО «Альбион», Новосибирск). Корм (SNIFF, Германия) и воду давали без ограничений. Корм и подстилку предо- ставляли животным после автоклавирования (121 °С). Протокол эксперимента одобрен комиссией по биоэтике ИЦиГ СО РАН.

**Группы животных**

Исследования выполнены на потомках обоего пола линии CD1 в возрасте 3–12 нед., полученных с использованием методов ВРТ. В соответствии с методом получения ис- следованные животные разделены на четыре группы:

- потомки матерей, не подвергавшихся эксперимен- тальным воздействиям в период беременности (кон- троль). Животных исследовали в возрасте 3 (n = 113), 7 (n = 113), 10 (n = 111) и 12 (n = 16) нед.;
- потомки, полученные после культивирования in vitro двухклеточных эмбрионов до стадии бластоцист и пересадки самкам-реципиентам (2 кл. – бл.). Животных исследовали в возрасте 3 (n = 23), 7 (n = 23), 10 (n = 19) и 12 (n = 16) нед.;
- потомки, полученные путем вымывания эмбрионов на стадии бластоцисты и пересадки самкам-реципиентам (Бл. – бл.). Животных исследовали в возрасте 3 (n = 30), 7 (n = 19), 10 (n = 19) и 12 (n = 16) нед.;
- потомки, полученные путем вымывания эмбрионов на стадии двух клеток и пересадки самкам-реципиентам (2 кл. – 2 кл.). Животных исследовали в возрасте 3 (n = 27), 7 (n = 27), 10 (n = 27) и 12 (n = 16) нед.

## Экспериментальные процедуры

**Вазэктомия самцов.** Самцам в возрасте 8–10 нед. произ- водили вазэктомию путем пережигания семявыносящих канальцев. Процедура проводилась под общей анестезией (домитор 15 мг/100 г веса мыши, золетил 3 мг/100 г веса мыши).

**Подготовка самок-доноров эмбрионов.** Для стимуля- ции овуляции проводили процедуру суперовуляции самок, которую выполняли в два этапа. На первом этапе самкам за 2 ч до выключения света (18:00 по местному времени) вводили внутрибрюшинно по 5 IU гонадотропина сыво- ротки жеребых кобыл (PMSG) (Intervet International B.V., Нидерланды). На втором этапе, через 48 ч после введения PMSG, этим же самкам внутрибрюшинно вводили по 5 IU человеческого хорионического гонадотропина (hCG) (Intervet International B.V., Нидерланды). Сразу после вве- дения hCG самок по одной подсаживали к фертильным самцам той же линии и утром следующего дня самок про- веряли на наличие вагинальных пробок. Через 24 ч после обнаружения вагинальной пробки извлекали яйцеводы и с помощью шприца вымывали двухклеточные эмбрионы. Через 3 сут выделяли матки, из которых вымывали бла- стоцисты. Вымытые эмбрионы помещали в каплю среды HTF (human tubal fluid). Эмбрионы с нормальной морфологией переносили в заранее подготовленную кап-лю среды KSOM, покрытую минеральным маслом, и помещали в CO2-инкубатор (37 °С) до трансплантации эмбрионов псевдобеременным самкам-реципиентам.

**Индукция псевдобеременности у самок-реципиен- тов эмбрионов.** Перед выключением света в комнате содержания животных (17:00) в клетку с изолированно содержащимся вазэктомированным самцом подсаживали трех самок. Утром следующего дня самок проверяли на наличие вагинальных пробок. Самок с вагинальными пробками отсаживали в отдельные клетки.

**Культивирование эмбрионов.** Часть двухклеточных эмбрионов, полученных описанном выше методом, в те- чение 3 дней инкубировали в культуральной среде KSOM АА при 5 % CO_2_ и 37 °С. Бластоцисты без морфологиче- ских дефектов пересаживали самкам-реципиентам

**Пересадки эмбрионов.** Самок-реципиентов двухклеточных эмбрионов (12 ч после подсадки самок к вазэк- томированному самцу) и бластоцист (3–3.5 сут после подсадки самок к вазэктомированному самцу) усыпляли при помощи ингаляционного наркоза – изофлурана (Baxter, США). Наркотизированным самкам подсаживали двухклеточные эмбрионы через воронку в яйцевод, а бластоцисты – в матку через надрез со стороны спины. После подсадки 8–10 эмбрионов самок отсаживали в индиви- дуальные клетки и содержали одиночно на протяжении беременности и выкармливания.

## Исследование потомков

**Взвешивание потомков.** Потомков взвешивали в возрас- те 3 (при отъеме от матерей), 7 и 10 нед.

**Определение состава тела.** Измерения общего жира и тощей массы проводили половозрелым потомкам в воз- расте 7 и 10 нед. при помощи низкопольного магнитно- резонансного томографа (EchoMRI, США).

**Глюкозотолерантный тест.** Толерантность к глюкозе исследовали у потомков в возрасте 11–12 нед. За 16 ч до инъекций глюкозы из клеток содержания мышей из- влекали кормушку. Глюкозу («ПанЭко», Россия) вводили внутрибрюшинно из расчета 10 мкл 20 % глюкозы на 1 г веса мыши. Кровь брали из кончика хвоста в 5 временных точках: 0 – базовый уровень глюкозы до введения и по- сле, 1 – через 15 мин, 2 – через 30 мин, 3 – через 60 мин и 4 – через 120 мин. Измерение уровня глюкозы проводили с помощью глюкометра Contour TS (Bayer, Швейцария). В качестве интегрального показателя глюкозотолерантно- го теста (ГТТ) рассчитывали площадь под кривой концен- трации глюкозы (average under curve – AUC).

**Статистический анализ**

Проверка на нормальность распределения эмпирических данных показала, что для всех изучаемых параметров можно использовать параметрическую статистику. Меж- групповые сравнения средних проводили с помощью однофакторного дисперсионного анализа, определяя наименьшую достоверную разность (least significant difference – LSD). Статистическую зависимость показателей фе- нотипа от числа новорожденных и массы тела при отъеме от матерей оценивали на основе линейных корреляций. Влияние вариантов пересадки и пола потомков на массу и состав тела анализировали путем двухфакторного ковари- ационного анализа (ANCOVA) с факторами «пол» и «ва- риант пересадки» и ковариатами «число новорожденных» и «масса тела» при отъеме. Для глюкозы и показателей теста толерантности к глюкозе применяли двухфакторный дисперсионный анализ (ANOVA).

## Результаты

**Плодовитость и масса тела при отъеме от матерей**

На размер пометов при отъеме от матерей значимо влиял способ их получения (табл. 1). При пересадках эмбрионов отмечено существенное снижение размера пометов по сравнению с контрольной группой. Вместе с тем разные способы пересадок не влияли на среднее число новорож- денных (p > 0.05, LSD-тест, см. табл. 1). Соотношение полов при разных вариантах пересадок и в целом по всем экспериментальным группам хотя и было сдвинуто в пользу самцов (45 самцов, 35 самок), но этот сдвиг не был статистически значимым: χ^2^ = 0.63, p = 0.43 при сравнении с теоретически ожидаемым 1:1. Ковариационный анализ (ANCOVA) изменчивости массы тела трехнедельных по- томков с факторами «группа», «пол» и размером помета в качестве ковариаты показал значимые эффекты группы: F_3.184_ = 11.42, p < 0.001. Фактор пола потомка, а также вза- имодействие факторов группы и пола новорожденных не влияли на массу потомков в этом возрасте: F_1.184_ = 0.671, p = 0.414 и F_3.184_ = 0.597, p = 0.618 соответственно. При сравнении массы трехнедельных потомков, полученных при разных вариантах пересадок, отмечено, что самцы и самки в группах 2 кл. – бл. были самыми тяжелыми, а в 2 кл. – 2 кл. – самыми легкими (см. табл. 1).

**Table 1.**
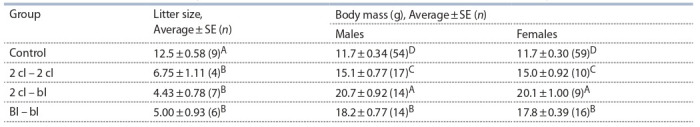
The size of the litter and body mass when weaned in mice obtained at different variants of embryonic transfers Note. Group designations here and beyond: Control – descendants derived from natural mating; 2 cl – 2 cl – descendants obtained by washing embryos at the stage of two cells and transplantation to female recipients; 2 cl – bl – descendants obtained after in vitro cultivation of two-celled embryos to the stage of blastocysts and transplantation to female recipients; Bl – bl – descendants obtained by washing embryos at the blastocyst stage and transplantation to female recipients. A, B, C, D – different letters indicate significantly different arithmetic means (p < 0.05, LSD-тест); SE – standard error; n – number of animals in the group.

Размер помета и масса тела в возрасте 3 нед. коррели- ровали с массой тела, относительным содержанием жира и тощей массой потомков в возрасте 7 и 10 нед. (табл. 2), но не коррелировали с концентрацией глюкозы и пока- зателями ГТТ. Исходя из этих результатов, все варианты межгрупповых сравнений массы и состава тела выпол- няли с помощью ковариационного анализа (ANCOVA), в который наряду с анализируемыми факторами включали две ковариаты – «размер помета» и «вес потомков» при отъеме от матерей.

**Table 2.**
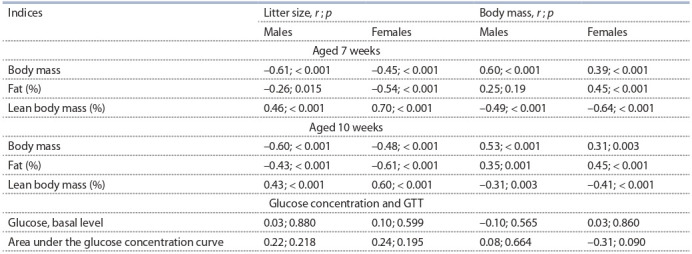
Correlation of body composition and glucose tolerance test with litter size and body mass of mice when weaned (combined sample data) Note. r – correlation coefficient, p – significance level.

## Масса и состав тела половозрелых потомков

Ковариационный анализ (ANCOVA) показал значимое влияние факторов группы, пола и их взаимодействия на массу и состав тела потомков в возрасте 7 нед. (рис. 1). Статистическую значимость отличий самцов и самок каждой экспериментальной группы от контрольной группы также оценивали с помощью ANCOVA. У самцов в возрас- те 7 нед. установлено достоверное превышение контроль- ных значений массы тела и относительного содержания жира в группе 2 кл. – бл. Масса тела самок в этом возрасте в контрольной и экспериментальной группах значимо не различалась. Вместе с тем содержание жира у самок, полу- ченных при разных вариантах пересадок, достоверно пре- восходило значения, наблюдаемые у контрольных особей. Соответственно, тощая масса самок экспериментальных групп была ниже таковой в группе контроля. Различная фенотипическая реакция на пересадки самцов и самок подтверждена статистически значимыми эффектами взаи- модействия факторов «группа» и «пол» (см. рис. 1). Объ- единение в общую группу данных по всем пересаженным потомкам показало, что содержание жира и тощей массы статистически значимо отличалось от группы контроля только у самок: жир – 16.8 ± 0.54 против 12.5 ± 0.39 % ( p < 0.05, ANCOVA); тощая масса – 75.4 ± 0.60 против 81.9 ± 0.43 % ( p < 0.05, ANCOVA).

**Fig. 1.**
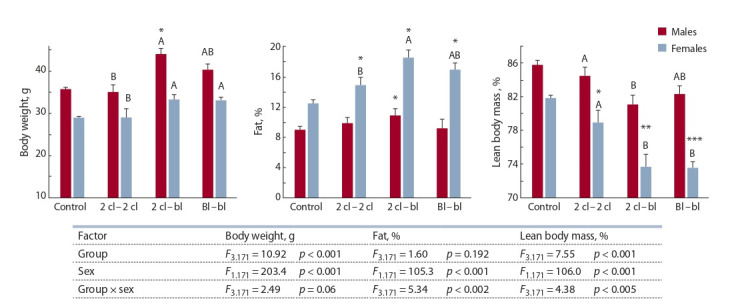
Body weight and relative values of fat and lean mass as % of body mass in the offspring of mice at the age of 7 weeks. Hereinafter the table shows the effects of the experimental group and the sex. ANCOVA with covariates (effects not shown) by the litter size and body weight at the age of 3 weeks. * p < 0.05; ** p < 0.01; *** p < 0.001 compared with control group. Different letters indicate significantly different arithmetic means ( p < 0.05, LSD test). ANCOVA calculated for males and females of each experimental group.

Поскольку размер помета достоверно не различался между экспериментальными группами, для выявления эффектов, обусловленных разными вариантами пере- садок, статистические сравнения между этими группами проводили отдельно для самцов и самок с помощью однофакторного дисперсионного анализа. Наибольшие значения массы тела установлены у особей обоего пола в группе 2 кл. – бл., наименьшие – в группе 2 кл. – 2 кл. (см. рис. 1). Относительное содержание жира, как и масса тела, были наибольшими у самцов и самок группы 2 кл. – бл., а значения тощей массы, соответственно, наименьшими.

Статистический анализ межгрупповых различий в воз- расте 10 нед., выполненный по изложенной выше схеме, показал значимое влияние экспериментальной группы, пола и взаимодействия факторов (группы и пола) на общую и тощую массу потомков (рис. 2). На содержание жира фактор «группа» не оказывал значимого влияния, но достоверно влиял фактор пола, а также взаимодей- ствие факторов – «пол» и «группа». При сравнении экс- периментальных групп с контрольной установлено, что у самцов доля жира превышала контрольные значения только в группе 2 кл. – 2 кл., а тощая масса была ниже, чем в контроле, в группах 2 кл. – бл. и Бл. – бл. У самок всех экспериментальных групп относительное содержание жира превышало контрольные значения. Соответственно, тощая масса была ниже, чем в контроле, но статистически значимым это различие было только в группе Бл. – бл. Как и в возрасте 7 нед., изменение состава тела, обуслов- ленное пересадкой эмбрионов, было более выраженным у самок, чем у самцов. При объединении данных групп потомков разных вариантов эмбриональных пересадок статистически значимые отличия от контрольной группы были выявлены только у самок: жир – 19.5 ± 0.65 против 13.4 ± 0.46 % ( p < 0.05, ANCOVA); тощая масса – 71.8 ± 0.65 против 77.5 ± 0.46 % ( p < 0.05, ANCOVA).

**Fig. 2.**
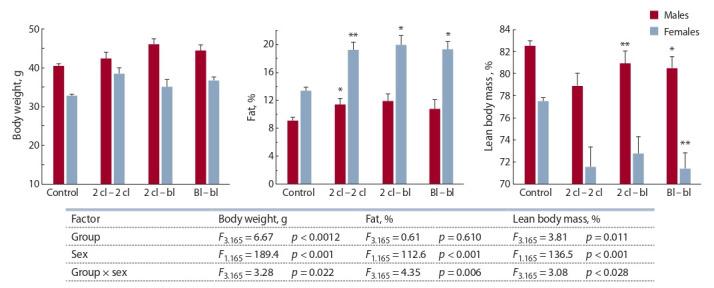
Body weight and relative values of fat and lean mass as % of body mass in the offspring of mice at the age of 10 weeks. * p < 0.05; ** p < 0.01 compared with control group. ANCOVA with covariates by the litter size and body weight at the age of 3 weeks.

Экспериментальные группы обоего пола между собой достоверно не различались. Эти результаты показывают, что если влияние способа получения потомков на массу тела нивелируется в возрасте 10 нед., то различия в жире и тощей массе по сравнению с группой контроля сохра- няются и в этом возрасте.

## Глюкозотолерантный тест

Уровень глюкозы до и во время нагрузочной пробы (ГТТ) не коррелировал с числом потомков в пометах и массой тела при отъеме от матерей. Это обстоятельство позволило проанализировать влияние пересадок и пола, не прибегая к использованию ковариат. Двухфакторный дисперси- онный анализ показал, что базальный уровень глюкозы существенно зависел от пола потомков (F1.56 = 19.74, p < 0.001) и взаимодействия факторов «пол» и «группа» (F3.56 = 3.69, p = 0.017) (табл. 3). Собственный эффект экс- периментальной группы был статистически незначимым (F3.56 = 1.90, p = 0.14). Концентрация глюкозы в разные сроки нагрузочной пробы (ГТТ) была достоверно ниже у самок разных групп, чем у самцов (рис. 3). Варианты пересадок статистически значимо влияли на уровень глю- козы, измеренный на 30-й мин ГТТ. При этом наибольшие значения отмечены у контрольных особей, а наименьшие – у потомков группы 2 кл. – бл.

**Fig. 3.**
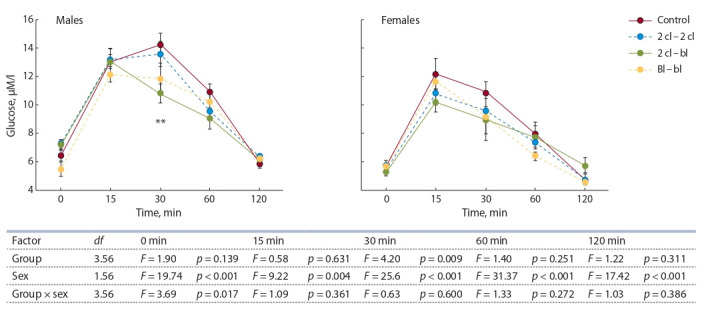
Glucose concentration after carbohydrate load. The table below shows the effect of the experimental group and sex on glucose concentration at different times after carbohydrate load. ** p < 0.01 compared with control group (Student’s t-test).

**Table 3.**
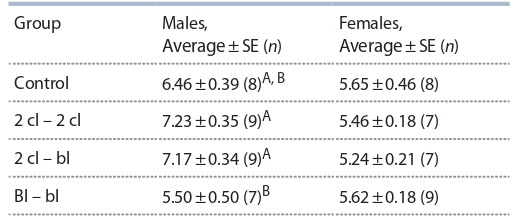
Basal glucose levels in mature male and female mice obtained at different variants of embryonic transfer ^A, B^ – different letters indicate significantly different arithmetic means (p < 0.05, LSD test); SE – standard error, n – number of animals in the group.

Площадь под кривой прироста концентрации глюкозы (AUC) варьировала в зависимости от пола животных и вза- имодействия факторов пола и группы (рис. 4). Последнее обстоятельство выражалось в том, что варианты пересадок влияли на AUC только у самцов. При этом наибольшие значения AUC отмечены у контрольных животных и по- томков, полученных при вымывании и пересадке бластоцист (группа Бл. – бл.). У самцов групп 2 кл. – 2 кл. и 2 кл – бл. значения AUC были достоверно ниже.

## Обсуждение

Результаты наших исследований показали, что пересадка эмбрионов суррогатной матери даже без предварительной инкубации оказывает долгосрочное влияние на метаболический фенотип взрослых потомков. Эти данные под- тверждают положение о высокой вероятности эпигенети- ческого перепрограммирования на доимплантационной стадии развития (Fleming et al., 2004; Rehfeldt, Kuhn, 2006; Burdge et al., 2007; Nelson, Lawlor, 2011; Fleming et al., 2012; Mulligan et al., 2012). Следует отметить, что в по- давляющем большинстве исследований долговременных последствий ЭКО в качестве главного фактора рассматри- вают состав культуральной среды (Fernández-Gonzalez et al., 2004; Dumoulin et al., 2010; Bouillon et al., 2016). Наше исследование показывает, что влияние культивирования эмбрионов in vitro от двух клеток до бластоцисты проявля- ется главным образом в большей массе тела и содержании жира у самцов в возрасте 7 нед. по сравнению с самцами, полученными естественным способом. В отличие от сам- цов масса тела самок, полученных при разных вариантах пересадок, не отличалась от группы контроля в возрасте 7 и 10 нед. Однако самки экспериментальных групп имели большее, чем в группе контроля, относительное содержание жира и меньшие значения тощей массы. Поскольку эти же отличия от контрольных особей были статистиче- ски достоверными как в экспериментах с культивирова- нием эмбрионов от двух клеток до бластоцист, так и при пересадках двух клеточных эмбрионов и бластоцист без культивирования, можно заключить, что сама процедура эмбриотрансплантации вносит вклад в метаболические изменения потомков женского пола.

Кроме того, условия преимплантационного этапа и ста- дия развития в момент пересадки влияли на толерантность к глюкозе потомков мужского пола, полученных после пересадок двух клеточных эмбрионов и бластоцист и по- сле культивирования in vitro. При этом следует отметить, что самцы, рожденные после пересадок интактных бла- стоцист, не отличались по этому признаку от контрольных особей. Полученные в нашем исследовании данные массы тела и толерантности к глюкозе демонстрируют некоторую схожесть долговременных последствий культивирования на преимплантационной стадии развития с метаболиче- скими изменениями у взрослых потомков (Donjacour et al., 2014; López-Cardona et al., 2015). Анализ данных ли- тературы о влиянии ВРТ на пре- и постимплантационное развитие, а также постнатальный фенотип показывает, что воздействие ВРТ зависит от вида млекопитающих, пола, используемой культуральной среды, а также возрас- та модельных видов (Duranthon, Chavatte-Palmer, 2018). При этом авторы подчеркивают, что очень трудно сделать общие выводы, за исключением того факта, что процедуры ВРТ влияют на липидный обмен и метаболизм глюкозы. Предложено несколько связанных между собой гипотез, объясняющих, каким образом неблагоприятные факторы на ранних этапах развития могут оказывать долгосрочное влияние на здоровье потомков (Feuer, Rinaudo, 2012, 2017).

Гипотеза начального триггера предполагает, что в ос- нове относительно сходных метаболических фенотипов (метаболизм глюкозы, липидный обмен и изменения ар- териального давления) лежат эпигенетические механизмы программирования генома. Период развития от стадии зиготы до бластоцисты является наиболее эпигенетически уязвимым, поэтому считается, что реакция эмбриона на искусственную среду приводит к изменениям экспрессии генов, которые включены в программу формирования обмена веществ. Согласно следующей гипотезе, влияние манипуляций in vitro на экспрессию ключевых генов, выполняющих функции регулятора транскрипции в пре- димплантационных эмбрионах, может по-разному влиять на типы клеток и объяснить специфичность профилей экспрессии генов в разных тканях, наблюдаемых при использовании ВРТ. Наконец, предполагается влияние условий преимплантационного развития на глобальную экспрессию генов, обусловленное транскрипционными изменениями, которые эпигенетически поддерживаются в нескольких локусах, в том числе при дифференцировке клеток (Feuer et al., 2014).

Следует отметить, что значительно меньше внимания уделяется долговременным последствиям ВРТ, обуслов- ленным реакцией суррогатной матери на эмбриональные антигены, формирующей гуморальное обеспечение для нормального развития плодов. Сегодня уже нет никаких сомнений, что материнские Т- и В-клетки распознают антигены плода и реагируют на его присутствие. Это явление подтверждено на экспериментальных моделях, в которых ответы Т- и В-клеток на природные или модель- ные антигены прослеживались in vivo и in vitro (Moldenhauer et al., 2010; Taglauer et al., 2010). Кроме того, наличие антиген-реактивных Т-лимфоцитов и антител к генам гистосовместимости хорошо известно и задокументировано у беременных женщин (James et al., 2003; Kahn, Baltimore, 2010). Результаты наших ранее опубликованных исследований показывают долговременные эффекты иммуногенетических различий между суррогатной матерью и эмбрионом на стадиях двух клеток и бластоцисты на метаболический и поведенческий фенотип взрослых потомков (Gerlinskaya, Evsikov, 2001; Gerlinskaya et al., 2019). Влияние факторов иммуногенетического диалога матери и плода на фенотип потомков может отличаться при пересадках, выполняемых на аутбредных (в нашем случае линия CD1) и инбредных линиях мышей.

## Заключение

Таким образом, развитие доимплантационных эмбрионов in vitro и даже их пересадки без предварительного культи- вирования влияют на метаболический фенотип потомков. Эти ключевые процедуры ВРТ могут существенно влиять на воспроизводимость результатов при использовании технологий криоархивирования для воспроизводства генетических линий лабораторных животных, хозяйствен- но значимые признаки и репродукцию ценных пород сельскохозяйственных животных, а также вероятность метаболических отклонений у новорожденных, зачатых с помощью экстракорпорального оплодотворения

## Conflict of interest

The authors declare no conflict of interest.
